# Direct tuning of soliton detuning in an ultrahigh-*Q* MgF_2_ crystalline resonator

**DOI:** 10.1515/nanoph-2023-0325

**Published:** 2023-09-11

**Authors:** Heng Wang, Bing Duan, Kai Wang, Xing-Yu Wu, Yong-Pan Gao, Bo Lu, Daquan Yang, Chuan Wang

**Affiliations:** School of Science, Beijing University of Posts and Telecommunications, Beijing, China; School of Information and Communication Engineering, Beijing University of Posts and Telecommunications, Beijing, China; School of Artificial Intelligence, Beijing Normal University, Beijing, China

**Keywords:** soliton microcombs, ultrahigh-*Q*, crystalline resonator

## Abstract

The dissipative Kerr soliton combs based on microresonators have attracted wide attention due to their high coherence and on-chip integration. Meanwhile, the soliton microcombs have shown broad applications in coherent communication, on-chip low-noise microwave synthesizer, optical clock, etc. However, the performance of these applications is typically limited by their bandwidth as the precise tuning of the soliton microcombs usually relies on the thermoelectric cooler, which is slow and may increase the system’s complexity. Here, we demonstrate the observation of dissipative solitons based on the magnesium fluoride resonator with an ultrahigh-quality (*Q*) factor of about 927 million. The ‘power-kicking’ scheme is employed to lock and stabilize the solitons actively. Also, tuning the acousto-optical modulator allows changing the bandwidth and recoil of the solitons. This approach enables more direct and concise feedback and reduces the system’s complexity.

## 1 Introduction

Optical frequency combs (OFC) based on resonators can feature high phase and frequency stability which has gained wide attention and rapid developments, in both fundamental science and technology. In particular, the dissipative Kerr soliton (DKS) in optical resonator corresponds to a broadband frequency comb that leads to a variety of applications in coherent optical communications [1], optical clocks [2], ultrafast distance measurement [3], dual-comb spectroscopy [4], ultra-low noise microwave synthesizer [5–8], to name a few. Recently studies have centered on generating soliton microcombs in resonators with different materials, such as silicon dioxide (SiO_2_), silicon nitride (Si_3_N_4_), lithium niobate (LiNbO_3_), and magnesium fluoride (MgF_2_) [9–12]. Especially, for MgF_2_, with its high nonlinearity from UV to mid-IR bands, lower thermo-optical coefficient, and smaller material absorption, is considered to be the ideal platform for stable soliton microcombs generation. It is worth noting that although the soliton microcombs have made great progress in many fields, there are still many fundamental limits on the effective tuning and optimizing the parameters of the soliton microbomb, such as the soliton bandwidth, and the soliton recoil, etc.

The thermal effects provide an approach to control the resonance mode of a resonator which has been widely used for direct access and tuning of the soliton microcombs. For example, the auxiliary laser is used to compensate for power changes in the soliton regime to generate soliton microcombs [13, 14], and another route is to use the integration of a thermoelectric cooler (TEC) under the resonator substrate to directly pump the soliton microcomb at a specific repetition rate and tune their bandwidth and recoil [15, 16]. Flexible switching of the repetition rate is realized by selecting the appropriate mode family [17]. However, the approach using thermal effects usually exhibits delayed feedback and additional power consumption. In addition, broader bandwidth tuning of soliton spectra span and high sensitivity gas sensing was achieved by altering the Fermi level of the graphene-based resonator to successively modulate its second- and higher-order chromatic dispersions [18–20]. The microcircuit system composed of integrated piezoelectric actuators also provide additional method for tuning the mode resonance which is very attractive for initiating, tuning, and stabilizing the soliton microcombs [21], but the above schemes usually require high fabrication technology and cost, and its comb frequency interval of hundreds of GHz is still a regret for microwave generation in X- (10 GHz) and K-bands (20 GHz) [22, 23]. Thus, there are still challenges to realize highly efficient and widely tunable soliton combs.

In this work, we demonstrated the active lock and direct tuning of soliton microcombs in an ultrahigh-*Q* MgF_2_ crystalline resonator by utilizing the ‘power-kicking’ scheme and servo feedback [24]. The MgF_2_ resonator with a diameter of about 2.6 mm is fabricated via fine polishing process, the *Q* factor approached 927.5 million [25, 26]. The single soliton comb with the repetition rate of about 26.5 GHz from 1520 nm to 1580 nm is generated when the pump power is around 23 dBm. We investigate the directly and rapidly tuning on the bandwidth and recoil of the soliton microcomb by controlling the modulation voltage and frequency of an acoustic–optic modulator (AOM). In addition, the smoothly switching from a static single soliton state to a breathing soliton state is achieved by continuously increasing the AOM modulation frequency, which facilitated easier access to the breathing soliton state for further studying its dynamics. Compared with the previous results using TEC, we only need to directly changing the modulation voltage and frequency of the AOM to obtain fast feedback of the soliton microcomb.

## 2 Device fabrication and the generation of solitons

**Figure 1:**
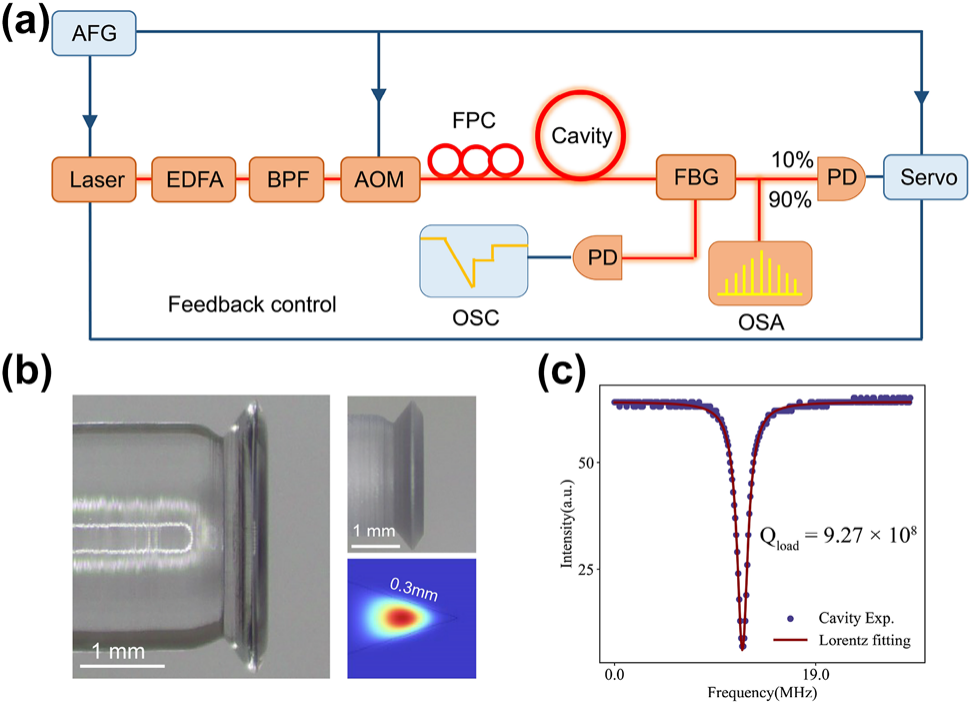
The setup and devices of for micorcombs generation. (a) Experimental setup for soliton microcombs generation. AFG, arbitrary function generator; EDFA, erbium-doped fiber amplifier; BPF, bandpass filter; AOM, acoustic–optic modulator; FPC, fiber polarization controller; FBG, fiber Bragg grating filter; PD, photodetector; OSC, oscilloscope; OSA, optical spectrum analyzer. (b) Optical microscope image of an MgF_2_ resonator with a diameter of about 2.6 mm. The insets on the right show images of a machined and unpolished resonator and the corresponding COMSOL simulation of the fundamental transverse magnetic mode profile. (c) The transmission spectrum of the MgF_2_ resonator and the quality factor of the mode was measured using a calibrated free spectral range (FSR) Mach–Zehnder interferometer (MZI) of approximately 927 million.

In this work, we show that the tuning of the pump would lead to the formation of dissipative solitons microcombs in resonators, the detailed setup is shown in Figure 1(a). The MgF_2_ crystalline resonator used here has a high-quality factor and anomalous dispersion. The fabrication process of the device could be realized as follows: first, the purchased magnesium fluoride cylindrical crystal is fixed on the motor spindle, which drives the crystal at high speed. And the crystal is rotated and shaped by using a custom diamond turning tool to estimate the edge geometry of the crystal resonator, which is typically simulated by COMSOL to ensure it has anomalous dispersion [25]. The resonator with the diamond turning process completed is shown in the inset in Figure 1(b), and the corresponding fundamental transverse magnetic mode profile is also presented. Then, in order to eliminate the cracks during mechanical shaping, the edges of the resonator need to be chemically polished using a diamond slurry, from large particles to small particles in succession. Especially, aiming to maintain the edge geometry, the size of the cracks on the crystal requires to be monitored real-time and the corresponding cracks need to be treated using diamond slurry with the right size particles until the edge surface is smooth. Finally, the resonator is cleaned using an ultrasonic cleaner to ensure that no diamond particles remained on the crystal surface. Figure 1(b) shows the MgF_2_ resonator that has completed the whole process, with a diameter of about 2.6 mm. Figure 1(c) illustrates the quality factor of this resonator, which approaches 927 million. Furthermore, its second-order group velocity dispersion is about −20.5 (fs^2^/mm).

Figure 1(a) shows the experimental setup used to generate the soliton microcombs, and here we use the ‘power-kicking’ scheme to actively lock the single soliton or multi-solitons [24]. The continuous output of the fiber laser is driven by the AFG for fine scanning and subsequently amplified by the EDFA and filtered by the BPF to remove noise from the EDFA. Then, the AOM is used to control the pump power into the resonator, modulated by the sine wave of the AFG. At the output, the power of the comb and the transmitted power are separated by the FBG. Meanwhile, the power of the microcomb is divided into two parts by the coupler, 90 % of the microcomb power is attenuated and recorded by the OSA, and the remaining part is monitored by the PD and sent to the servo control box. When the servo detects the voltage, an error signal is generated by subtracting the setpoint to adjust the pump frequency in feedback to maintain the average soliton power.

The basic principle of the ‘power-kicking’ scheme is to maintain the average soliton power by continuously tuning the pump frequency to change the pump detuning. Specifically, the pump detuning corresponds with the average soliton power of the resonator, and the expression could be expressed as follows [9, 24]:

(1) $$P_{\text{sol}}=\frac{2\eta A_{\text{eff}}}{n_{2}Q}\sqrt{-2n_{0}c\beta_{2}\delta \omega }$$

In Eq. (1), *η* denotes the coupling efficiency, and *n*_0_(*n*_2_) is the refractive(nonlinear) index. *A*_eff_ represents the effective mode area. *Q* is the quality factor, *c* is the light speed in a vacuum. The parameter $\beta_{2}=-n_{0}D_{2}/cD_{1}^{2}$ is the resonator second-order group velocity dispersion [27], and the *δω* = *ω*_0_ − *ω*_p_ is the cavity-pump detuning (*ω*_0_ and *ω*_p_ are resonant mode frequency and pump frequency, respectively).

**Figure 2:**
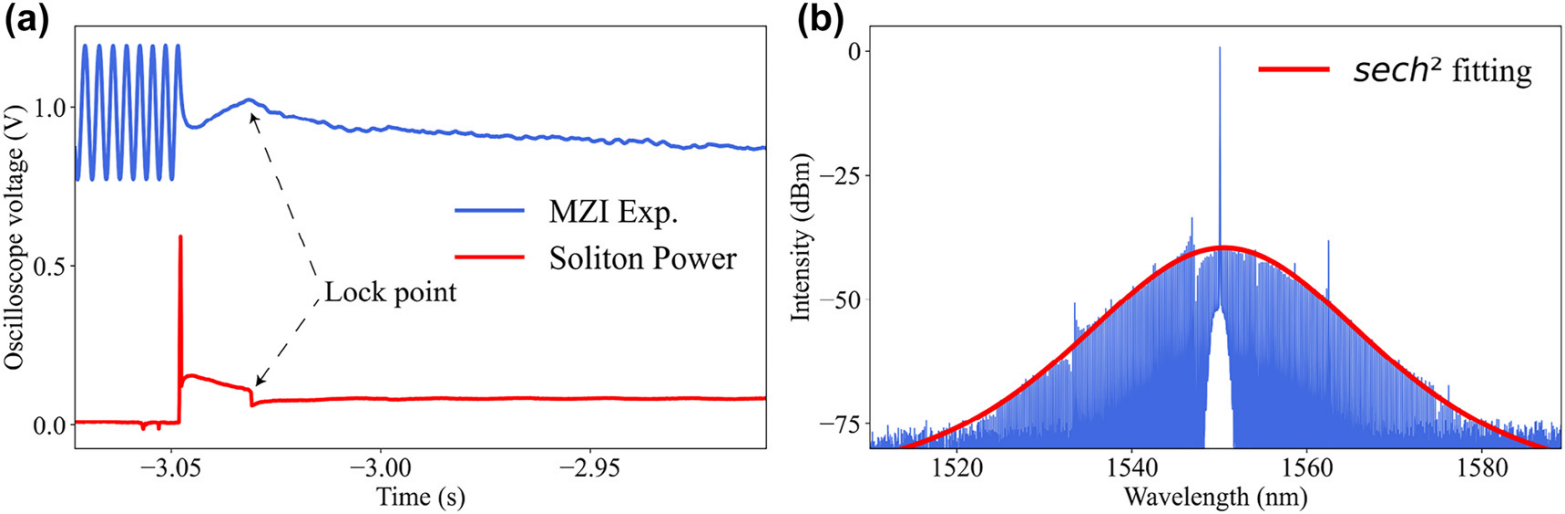
The power and spectrum of solitons. (a) Demonstration of the change in soliton power and pumping frequency when a single soliton state is locked. The blue line denotes the output power of MZI that is used to monitor the change in pump frequency, and the black arrow indicates the locking position. (b) The locked single soliton state, the red line is the *sech* ^2^ envelope fitting.

Figure 2(a) demonstrates the locking process of a single soliton, where the red line indicates the soliton power and the blue line is the output power of MZI, which is used as a reference for pump frequency changes. The process of locking mainly includes three steps, first, the laser frequency is tuned to the blue detuning of the resonant mode, thus the Turing state is generated. Subsequently, a carefully designed waveform is sent to the AFG to drive the AOM to rapidly reduce the pump power, which causes a blue shift of the resonant mode due to the Kerr nonlinearity and thermo-optical effects, thus leaving the pump frequency in the red-detuned soliton regime. The rising edge of the waveform then increases the pump power, extending the range of soliton presence. Unlike silica resonators, due to the smaller thermal effect of magnesium fluoride, the waveform can be designed more slowly to increase the lock success rate. Finally, to maintain the average soliton power, the servo box generates an error signal to change the pump frequency. In our experiments, the length of the locked single soliton step is about 80 μs. According to Eq. (1), the average soliton power is maintained, and the cavity pump detuning could be locked.

**Figure 3:**
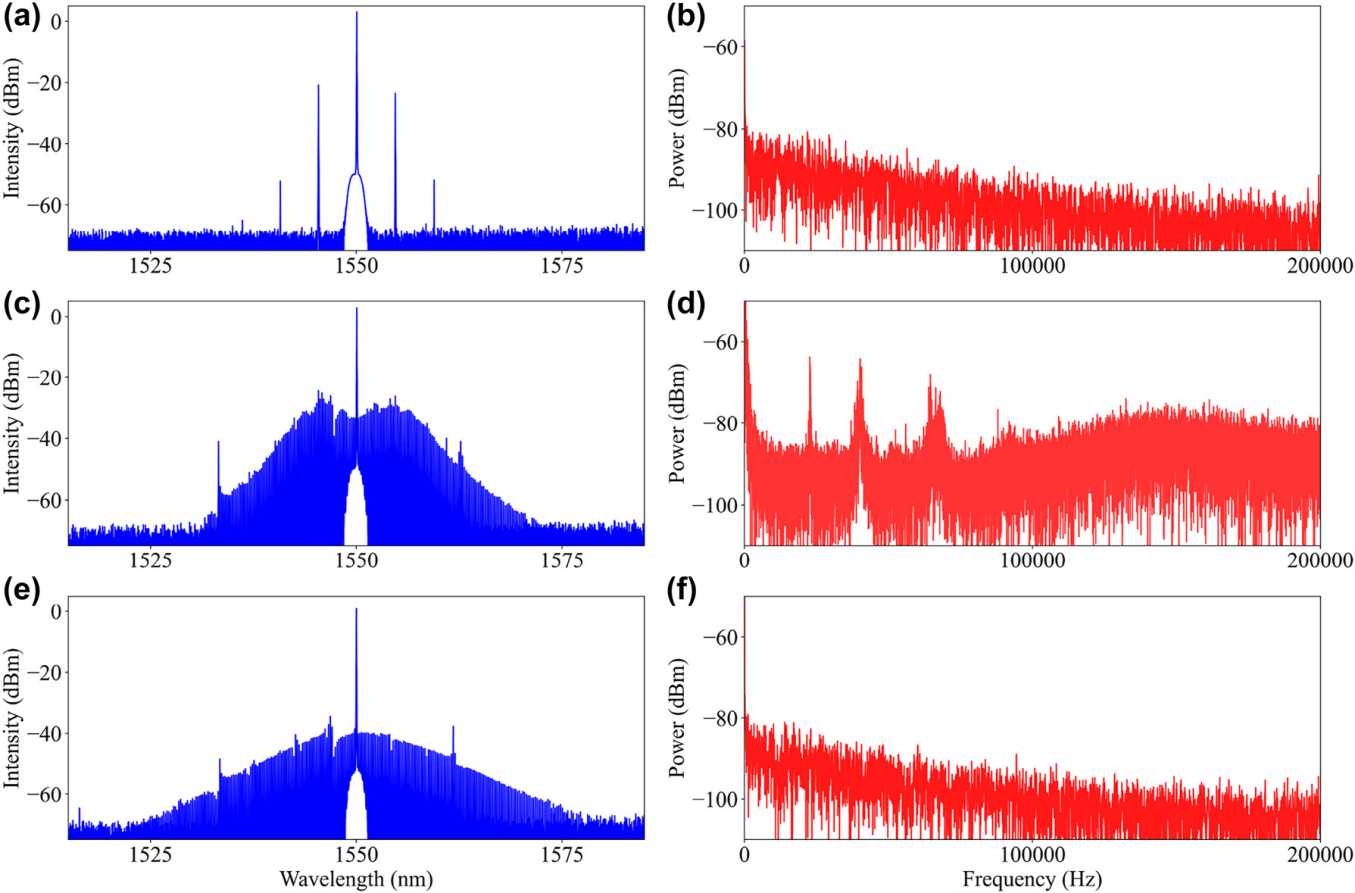
Spectral evolution of the soliton generation process with the corresponding low frequency RF. Turing state (a) and (b). MI state (c) and (d) and soliton state (e) and (f).

The spectral evolution during the soliton formation process can be divided into three stages, including a Turing state, the modulated instability (MI) state, and the static single soliton, as shown in the spectra and RF spectra in Figure 3. We detected low frequency RF evolution, where low frequency high-noise RF beat note was generated at the MI state due to the disordered waveform in the resonator. With the formation of a single soliton, the waveform in the resonator is ordered and the high-noise RF beat note disappears, and the corresponding low-noise beat RF beat note corresponds to the FSR of the resonator, which is beyond the detection range.

## 3 Results

### 3.1 Direct tuning of soliton microcombs

The MgF_2_ resonator shown in Figure 1(b) is used to generate a single soliton microcomb for direct spectral tuning. After activating the entire digital feedback loop, the soliton formation exists stably within the resonator as the thermal effects are compensated. Then, by expanding the upper and lower boundaries of the soliton power setpoint of the feedback program, we can slowly change the cavity-pump detuning by adjusting the AOM. The AOM acts as a controller that modulates the pump light intensity in the whole loop, and it is directly driven by the sinusoidal signal generated by the AFG to form the dynamic grating. When the pump light is incident on the acousto-optic medium, diffracted light will be generated due to the acousto-optic effect. Here, we directly adjust the signal parameters of the AFG, such as modulation voltage and modulation frequency, and we found the intensity of the diffracted light changes according to the variation of the loaded modulation signal.

**Figure 4:**
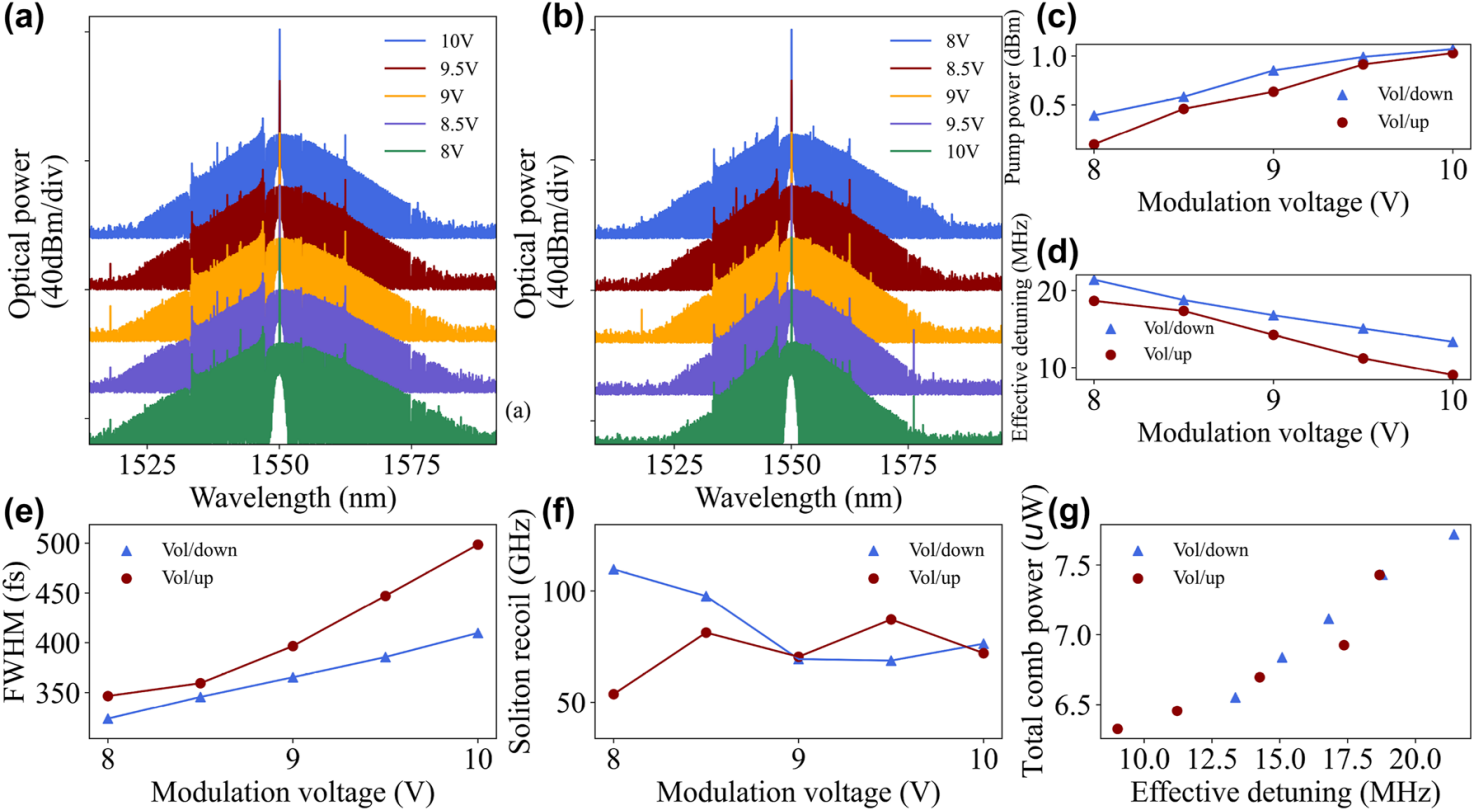
Spectrum evolution of the soliton with the AOM modulation voltage. (a) Variation of soliton spectrum when the AOM modulation voltage is gradually reduced from 10 V to 8 V. (b) Variation of soliton spectrum when the AOM modulation voltage is gradually increased from 8 V to 10 V. (c) The red dot ‘Vol/up’ indicates an increment in modulation voltage leading to the increase of pump power, and the blue dot ‘Vol/down’ indicates a decrement in modulation voltage leading to the decrease of pump power. (d) Increasing the modulation voltage leads to a decrease in the effective cavity-pump detuning. (e) and (f) Show the soliton pulse full-width at half-maximum (FWHM) and soliton recoil versus modulation voltage, the FWHM is derived from the *sech* ^2^ function fitting. The red and blue lines correspond to the spectral change diagrams (b) and (a), respectively. (g) Variation of total comb power (derived from a *sech* ^2^ function fitting) with effective cavity-pump detuning.

Firstly, we locked the soliton at a modulation voltage of 8 V and 10 V, respectively, and then gradually increased or decreased the voltage to 10 V or 8 V in a step of 0.5 V, while recording the intensity change of the pump light input to the OSA after attenuation. As shown in Figure 4(c), the characters ‘Vol/up’ or ‘Vol/down’ indicate the gradually increasing or decreasing of the modulation voltage, respectively. In the ‘Vol/up’ operation, the pump light intensity increases from 0.092 dBm to 1.03 dBm; while in the ‘Vol/down’ operation, the pump light intensity decreases from 1.071 dBm to 0.392 dBm.

In general, the tuning of the resonant frequency of optical resonators can be achieved by the thermal effects, namely the thermo-optical effect and the thermal expansion effect [28, 29]. The former leads to a change in refractive index (*n*), and the latter leads to a change in the photon roundtrip length (*L*) of the resonator. Specifically, the relationship between the temperature and the resonant frequency could be expressed as *ω* = *ω*_0_(1 − (*α*_*L*_ + *α*_*n*_)*δT*), here *ω* and *ω*_0_ are thermal shift and ‘cold’ cavity resonance frequencies, respectively. *δT* denotes the temperature change of the mode volume, which is positively related to the intracavity power. In this experiment, we noticed that the material of magnesium fluoride has a positive coefficient of thermal expansion *α*_*L*_ = (1/*L*)(∂*L*/∂*T*) and a thermo-optic coefficient *α*_*n*_ = (1/*n*)(∂*n*/∂*T*) [29]. This relation represents a red-shift of the frequency that an increase in intracavity power leads to a decrease in the cavity resonance frequency.

Figure 4(a) and (b) showed the changes in the spectral bandwidth of the soliton recorded during the ‘Vol/down’ and ‘Vol/up’ operations, respectively. During ‘Vol/up’ operation, the pump light is enhanced which heated the whole resonator. Thus, the thermal effect leads to a red-shift of the resonance frequency and a reduction in the cavity-pump detuning could be achieved. According to Eq. (1), when the detuning between the resonator and the pump is decreased, the average soliton power will decrease accordingly. The servo is used to detect the reduced voltage and drive the pump on the red-shift frequency band to maintain the set value of the average soliton power. In addition, it should be noticed that the average soliton power could also be affected by the local thermal effects of the resonator. For the microcomb system consisting of an MgF_2_ resonator and a silica tapered fiber, the thermo-optical effect can enhance the effective refractive index and coupling efficiency *η* as the temperature increases, meanwhile, the thermal expansion effect can affect the effective coupling length more efficiently, resulting in an enhancement of the coupling strength [16]. In other words, when the servo drives the laser to change the pump frequency, due to the increase of the coupling efficiency, the average soliton power can reach the setpoint with a relatively smaller detuning than before. In contrast, the solitons require a larger detuning to reach the setpoint in a ‘Vol/down’ operation. In fact, not only the average soliton power, but also the soliton pulse duration (FWHM) Δ*τ*_s_ (equivalent to the bandwidth of a soliton microcomb in the frequency domain) is related to the cavity-pump detuning, which could be expressed as [30]:

(2) $$\Delta \tau_{\text{s}}=\sqrt{\frac{-c\beta_{2}}{2n_{0}\delta \omega }}$$

As shown in Figure 4(d) and (e), the modulation voltage causes the variation of the cavity-pump detuning, which eventually leads to an increase or decrease in the soliton bandwidth. In fact, since the second-order group velocity dispersion of the resonator is almost constant, the soliton duration as Eq. (2) should be an inversely proportional function of the detuning.

As shown in Figure 4(f), we also observed the dynamical behavior of soliton recoil due to the narrow Raman gain bandwidth of the MgF_2_ material, which mainly stems from the avoidance of mode crossing leading to excessive power of certain combs, causing the spectral center of mass change [31–33]. As the modulation voltage changes, the excessive power of specific combs perturbs the center of mass of the spectrum, appearing to have no strong pattern in the change of recoil [34]. The total comb power evolves with the effective cavity-pump detuning, as shown in Figure 4(g), a larger detuning also implies a wider soliton bandwidth which brings higher total comb power.

**Figure 5:**
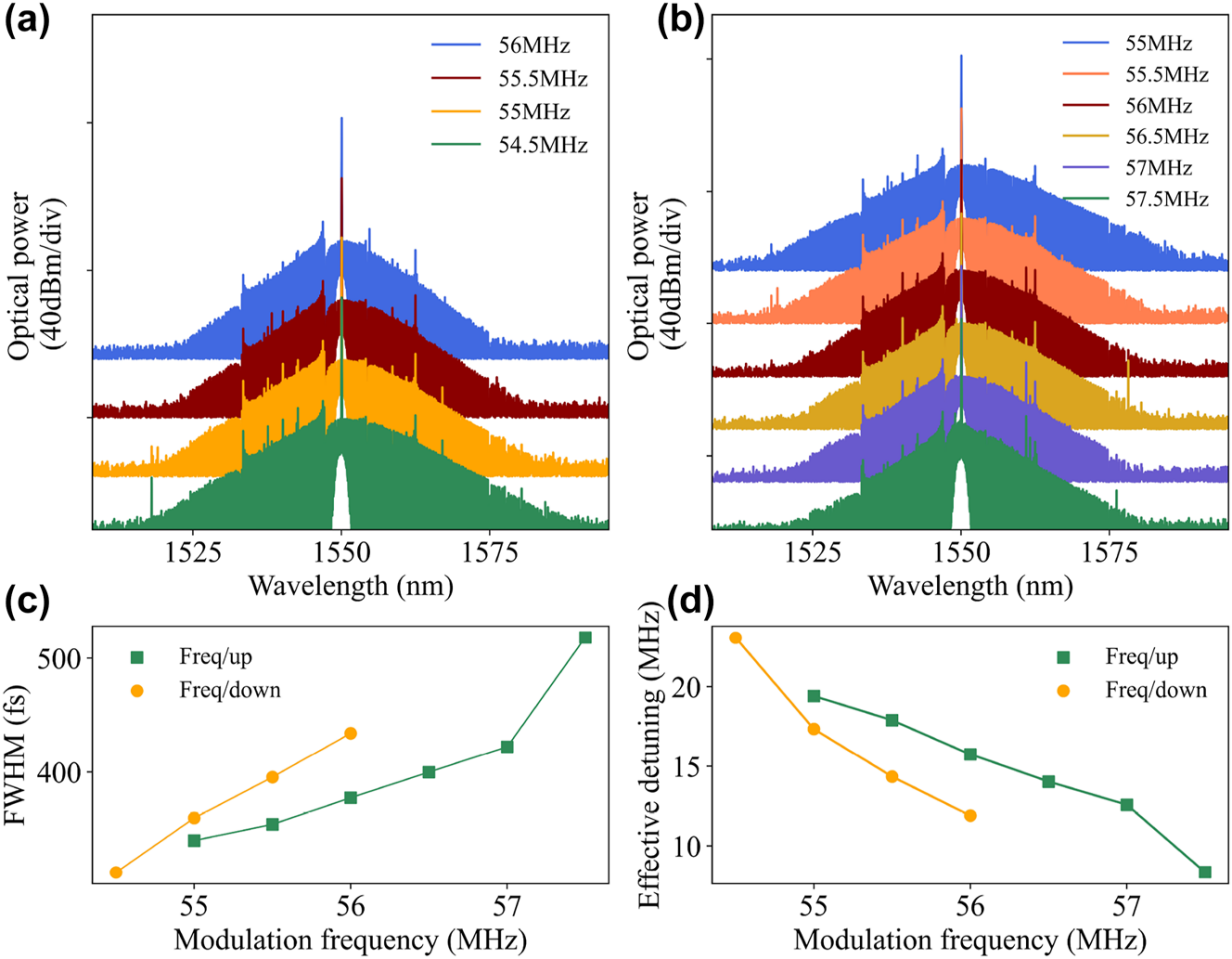
Spectrum evolution of the soliton with the AOM modulation frequency. (a) Variation of the soliton spectrum when the AOM modulation frequency is gradually reduced from 56 MHz to 54.5 MHz. (b) Variation of the soliton spectrum when the AOM modulation frequency is gradually increased from 55 MHz to 57.5 MHz. (c) Soliton pulses FWHM versus modulation voltage. (d) Increasing the modulation frequency leads to a decrease in the effective cavity-pump detuning.

Furthermore, the bandwidth of the soliton microcomb could be directly tuned by changing the modulation frequency of the AOM, keeping the modulation voltage constant. In our experiment, the solitons are locked at modulation frequencies of 56 MHz or 55 MHz, respectively, followed by a step of 0.5 MHz to gradually decrease or increase the modulation frequency. Figure 5(a) and (b) show the recorded soliton spectra with the gradually increasing and decreasing bandwidth, respectively. By measuring the intensity change of the pump light, we found that the pump power also gradually decreases when gradually decreasing the modulation frequency, leading to a decrease in the soliton duration (an increase in the frequency domain bandwidth), as shown by the orange dot in Figure 5(c). In the “Freq/up” operation, the increasing of soliton FWHM exhibits an obvious jump at the last 57.5 MHz, with a triangular shape in the spectrum. The spectral bandwidth and effective detuning are rapidly reduced (see the last green point in Figure 5(c) and (d)), which provides the possibility of direct access to the respiratory solitons.

### 3.2 Direct access to breathing soliton states

**Figure 6:**
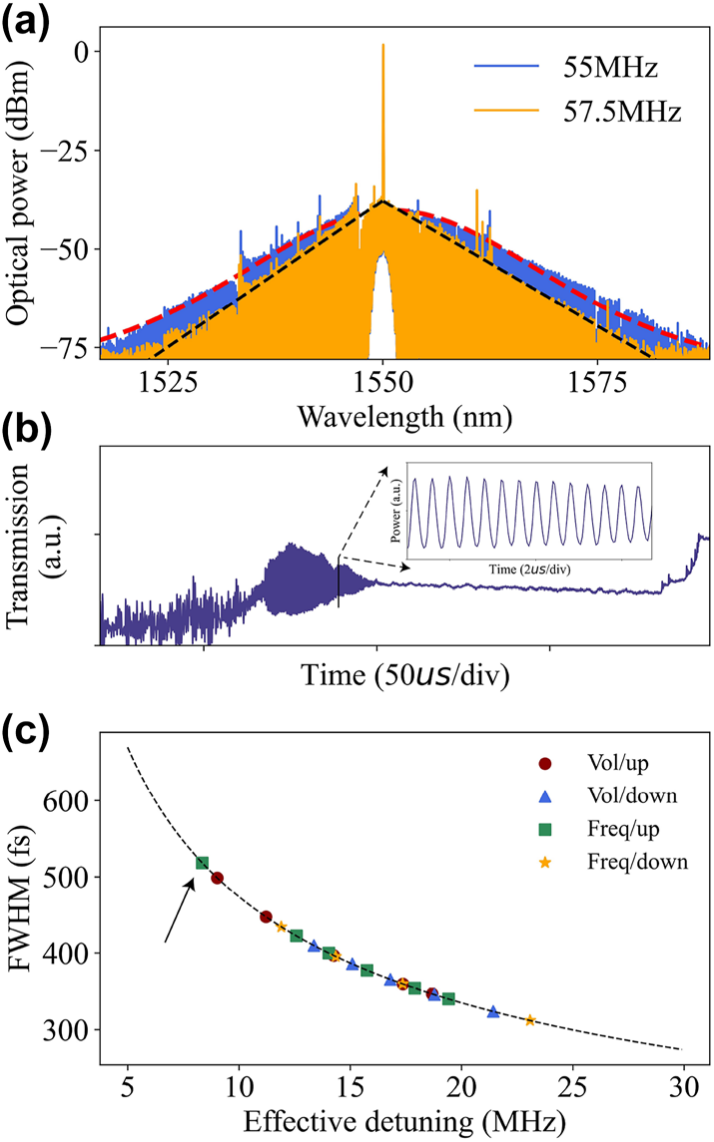
The spectrum properties of the soliton. (a) Breathing soliton spectrum of a triangular envelope (orange) and a static soliton spectrum (blue). (b) Resonator transmission spectrum, the inset shows the time-domain oscillations of the breathing soliton. (c) The measured soliton FWHM (derived from a *sech* ^2^ fit) is plotted versus the detuning (calculated from Eq. (2)), the black dashed line is the fitted curve.

In the experiment, we observed the transition from a stationary soliton state to a breathing soliton state (the spectrum clearly shifted to a triangular character) by continuously increasing the modulation frequency of the AOM so as to reduce the cavity-pump detuning. A continuous decrease in detuning leads to an increasing amplitude of oscillations, as in Figure 6(b), where the servo is unable to keep locking on to the breathing soliton, at which point re-increasing the detuning can re-stabilize the soliton. Distinct from the single static soliton we locked above, the breathing soliton exhibits periodic oscillation in both amplitude and pulse duration, resulting from the periodic energy exchange between the spectrum center and the wings, which is closely related to the Fermi–Pasta–Ulam recurrence [35]. This method of accessing breathing soliton states is the essence of the laser backward tuning method. When the AOM modulation frequency is increased to 57.5 MHz, the *sech* ^2^-shaped static soliton spectrum transforms into a triangular spectrum resulting from the averaging of the periodic broadening and compression of the comb bandwidth by OSA, as shown in Figure 6(a) [36, 37]. Figure 6(b) records the pump transmission when the pump laser scans linearly from short to long wavelengths, and the inset is a local magnification of the breathing soliton regime, showing rapid oscillations in the soliton power. It also illustrates that with the exception of the intermode breathing soliton [38], the breathing soliton regime is usually located at a relatively small pump detuning and is between the modulation instability and the static soliton regime so that accessing the breathing soliton state requires a continuously decreasing pump detuning while maintaining a high pump power.

In Figure 6(c), we demonstrate the relation of soliton FWHM versus the effective detuning when varying the modulation voltage and modulation frequency. It should be noted that the ‘Vol/up’ operation changes more soliton duration than the ‘Vol/down’ operation with the same 2 V modulation voltage. We speculate that this is related to the precision of the servo and the step position where it was initially locked. In order to increase the success rate of locking, we usually allow a certain error in the setpoint value, which can result in the servo not always locking to the same point consistently. And it is more difficult to continue to increase the detuning in a flatter area after locking a large detuning. At the same time, the existence range of the soliton is given by *δω*_max_ = *π* ^2^ *P*_in_/16*P*_th_, where *P*_in_ and *P*_th_ are the pump power and parametric oscillation threshold, respectively [39, 40]. Therefore, reducing the modulation voltage leads to a decrease in pump power, which results in a faster detuning of the pump frequency with the soliton existence range. Similarly, when decreasing the modulation frequency, the detuning increases, and the change in the bandwidth of the soliton spectrum is insignificant due to the flatter curve, and the pump will move out of the soliton existence range faster due to the smaller intracavity power and the shorter steps. Whereas when increasing the modulation frequency, it can be noticed from the green dot in Figure 6(c) that the smaller the detuning, the steeper the curve is, and the unit detuning brings about a larger change in the bandwidth, as in Figure 5(a) and (b), which will bring about a more pronounced change in the bandwidth. And by gradually increasing the modulation frequency, we can also directly access the breathing soliton state, corresponding to a detuning value of about 8.35 MHz (arrow pointing to green dot in Figure 6(c)).

## 4 Discussion

In this study, an MgF_2_ resonator with a diameter of about 2.6 mm and a *Q* factor close to 927.5 million has been fabricated by a precision polishing process. Based on the ‘power-kicking’ scheme, we experimentally demonstrate the soliton generation using the resonator and showed the modulation of the bandwidth of the soliton microcombs directly by using AOM. The scheme is direct and rapid, reducing the complexity of the comb system because TEC is not required. By varying the modulation voltage by 2 V, we change the soliton duration from 347 fs to 499 fs, corresponding to a change in detuning from 18.68 MHz to 9.03 MHz, for a total of about 152 fs in the “Vol/up” operation. In the “Vol/down” operation, the soliton duration was changed by about 86 fs, from 409 fs to 323 fs, corresponding to a detuning change from 13.37 MHz to 21.42 MHz. In the “Freq/up” operation, we change the soliton duration from 339 fs to 422 fs, which corresponds to a detuning change from 19.42 MHz to 8.35 MHz. In the “Freq/down” operation, we change the soliton duration from 434 fs to 311 fs, which corresponds to a detuning change from 11.42 MHz to 21.42 MHz.

We illustrated that the principle of the scheme is to increase or decrease the intracavity power by the AOM so that the pump detuning is changed, and then the new detuning is locked by the servo, which will change the soliton bandwidth. We also find that the resonator used for the experiment allows the static soliton to switch smoothly to the breathing soliton at detuning at about 8.35 MHz, enabling us to access the breathing soliton state directly.

This work provides a more immediate tuning scheme and a simple way to access the breathing soliton state for future microcombs systems. Power consumption and reliability are key issues that need attention in the tunable design of future microcombs systems, and the instability of the breathing soliton is something that should be avoided in stable working systems, so easy access is beneficial for subsequent studies.
